# Intentional Ingestion of Foreign Bodies: A Physician’s Agony

**DOI:** 10.7759/cureus.37677

**Published:** 2023-04-17

**Authors:** Siddharth Sankar Das, Suhasini Krishnan, Nisha K Mandhane, Husni S Shalak

**Affiliations:** 1 General Surgery, Dubai Hospital, Dubai, ARE; 2 Medicine, Dubai Academic Health Corporation, Dubai, ARE

**Keywords:** ingestion, object, foreign, recurrent, deliberate

## Abstract

Intentional foreign body ingestion is the phenomenon wherein one swallows a non-digestible object with the intent to cause self-injury. It is intentional in adult patients with a positive psychiatric history and can be a recurrent issue. Although the incidence of this condition is increasing, there are few existing articles on the subject that highlight its importance. This case report aims to present a unique patient encounter to emphasize the multispecialty approach required for management and provide an overview of the literature available on the subject regarding types of objects swallowed, selection of appropriate imaging modalities, and plans of management.

## Introduction

Deliberate ingestion of foreign bodies is a phenomenon frequently encountered by psychiatrists, emergency medicine physicians, surgeons, and gastroenterologists. Despite its increasing occurrence, there are few studies that highlight the need for a multispecialty approach to treatment [1]. Recurrent foreign body ingestion is most commonly seen in children, adults with psychiatric illnesses such as personality disorders, and prisoners [2]. The main difference is that in adults, ingestion of these objects is almost always intentional, while it is accidental in children [3]. Serious complications of foreign body ingestion include esophageal occlusion, bowel obstruction, perforation, necrosis, and fistula formation [4]. The most commonly used imaging modality in these cases is X-ray due to its accuracy in identifying radiopaque objects [2,4]. The majority of clinically stable patients will pass the object within four to five days, while for patients with complications, endoscopy and surgical intervention are required to retrieve the object [4].

## Case presentation

A 29-year-old Middle-Eastern male presented to the emergency department with complaints of severe abdominal pain in the epigastric region after ingesting multiple nails. The patient reported that earlier that day he was driving in a stressed and distractible state, following which he unknowingly poured his soft drink into a cup containing nails at the bottom. The patient was subsequently admitted to the hospital for close monitoring. His medical history was significant for cluster B personality disorder, intermittent explosive disorder, and epilepsy. His surgical history included a left laparoscopic pyeloplasty performed two years ago to correct hydronephrosis due to a congenital pelvic ureteric junction obstruction. His current medications included escitalopram 10 mg twice daily and mirtazapine 30 mg at bedtime. Although he had suffered from two bouts of hematemesis since admission, on examination, he was vitally stable and per abdomen soft with mild epigastric tenderness and brown stool per rectum. His lab investigations were within reference ranges.

**Figure 1:**
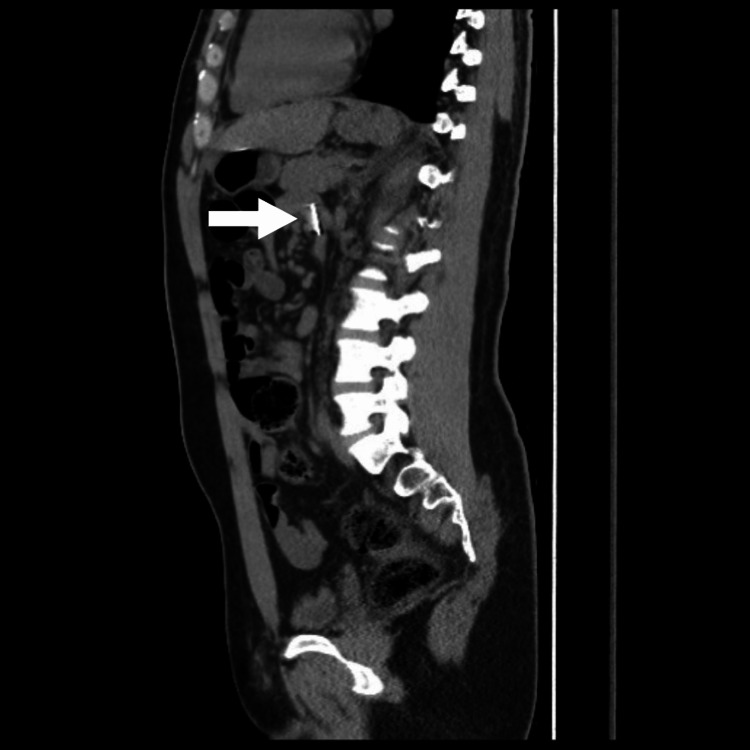
Computed tomography scan showing needle-shaped body in distal duodenum.

**Figure 2:**
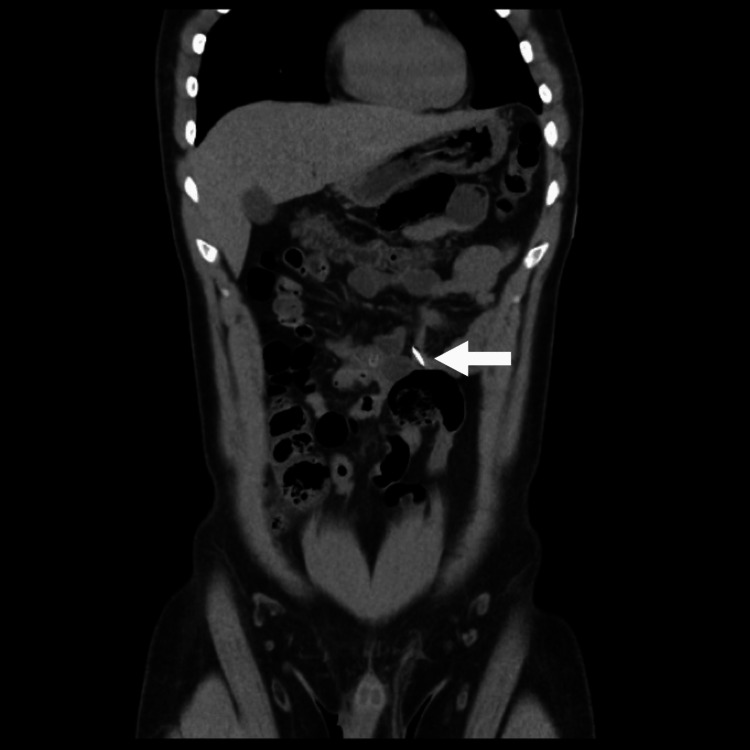
Computed tomography scan showing needle-shaped body in mid-jejunal loops.

**Figure 3:**
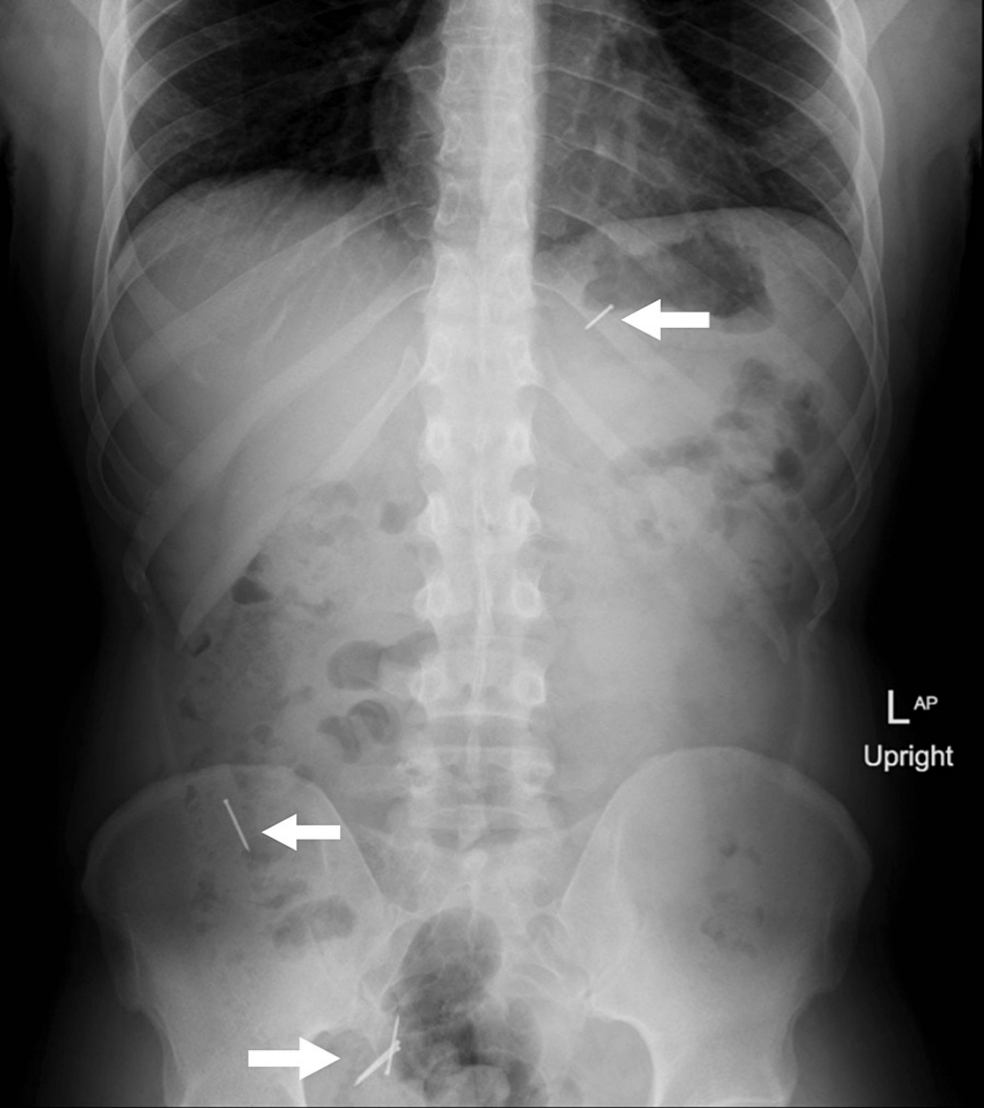
Abdominal X-ray showing five nails.

**Figure 4:**
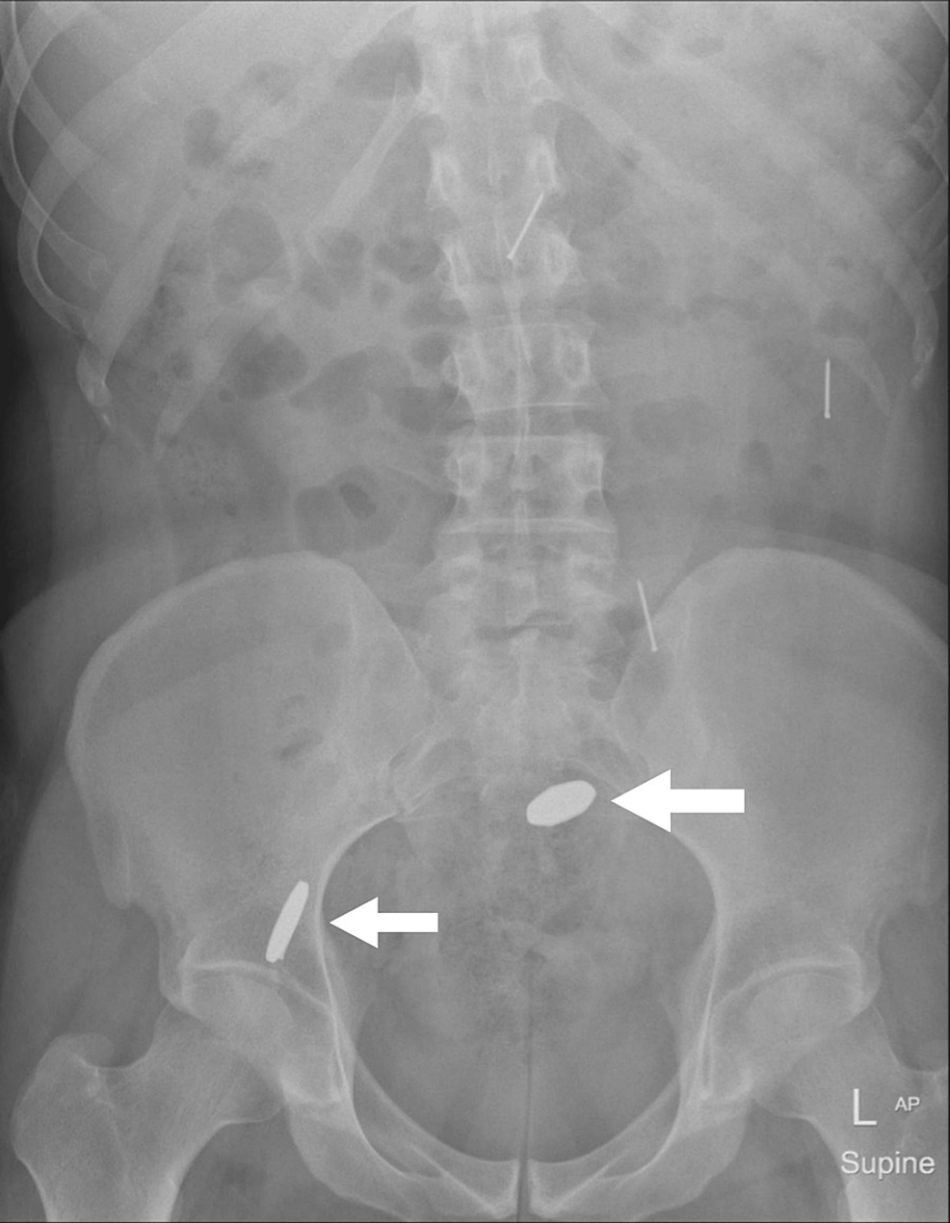
Abdominal X-ray showing two new coins.

Computed tomography (CT) scan without contrast showed approximately three linear needle-shaped foreign bodies noted in the second part of the distal duodenum, duodenojejunal junction, and mid-jejunal loops, as can be seen in Figure 1 and Figure 2. However, an abdominal X-ray showed five metallic-density foreign bodies (nails) in the pelvis, as shown in Figure 3. Follow-up serial X-rays revealed progression of the nails along the gastrointestinal tract as well as two new radiopaque foreign bodies, resembling coins, one in the left lumbar region at the level of the left transverse process of L3 and the second superior to the right acetabulum, as can be seen in Figure 4. There was no evidence of pneumoperitoneum or bowel obstruction. This indicated that the patient continued to ingest foreign substances even after admission to the hospital. Approval from the authorities was granted and his personal effects were searched for items easily swallowed or potentially dangerous. As the patient was clinically stable, surgical intervention was not required and he was continued on conservative management throughout his hospital stay. Endoscopy reported *Helicobacter pylori*-induced gastroduodenitis for which proton-pump inhibitor (PPI) infusion twice daily was prescribed. The plan of management was to continue conservative management with PPI infusion and advance the diet as would be tolerated the following day. The patient was also regularly reviewed by the psychiatrist, who monitored his condition and noted his improvement throughout his hospital stay.

Tracking the progression of the foreign bodies meant a prolonged hospital stay for the patient. Despite counseling him about his state and possible complications that could arise, he refused further investigations and requested to be discharged against medical advice. He followed up in the outpatient clinic a month later to report verbal confirmation of having passed the foreign bodies as well as the continuation of his psychiatric medications. Follow-up X-rays confirmed complete passage of the same.

## Discussion

Need for a multidisciplinary approach

What makes this case unique is the patient’s continued ingestion of the objects after hospitalization. This confirms self-injury, which is described as harming or punishing oneself deliberately [5]. It can be used as a means to seek medical attention. Intentional ingestion can be classified as a non-suicidal or parasuicidal subtype of self-injury, as the goal is to injure oneself without taking one’s own life [6]. Ghimire and colleagues report that the most common risk factors for repeated ingestion of foreign substances include male sex, imprisonment, and a positive psychiatric history, two of which were present in our patient [7]. Gitlin and colleagues note that this behavior is common to four major psychiatric illnesses, namely, psychosis, pica, malingering, and personality disorders, the last of which was positive in our patient [8]. According to Palese and colleagues, psychiatric patients engaging in these behaviors have histories of childhood neglect and sexual and/or physical abuse [6]. It is suspected that ingestion, as self-injurious behavior, can give patients a sense of control and power over their caregivers, as the issue is complex and usually requires a multispecialty approach to intervention, as evidenced in our patient who required consultation by multiple physicians [2,6]. It is important to involve other specialties, especially psychiatry, to effectively manage the associated ongoing psychiatric conditions for the medical or surgical treatment to progress and to prevent repeat occurrences [6].

Types of foreign bodies

The types of foreign bodies ingested by adult patients are usually small enough to be swallowed without difficulty and are easily accessible. Through successful identification of the ingested object, the level of urgency can be determined and the appropriate management can be initiated. One retrospective study identified 262 cases of foreign body ingestion in a single clinical setting and itemized a list of the objects once recovered, which included toothbrushes, stationery, cutlery, razor blades, and batteries [9]. Another study by O’Sullivan and colleagues revealed that batteries, sharp metals, and glass were the most commonly ingested materials among 36 cases of institutionalized and incarcerated patients [10]. A literature review conducted by Ambe and colleagues categorized the objects based on size, surface consistency, type of material, and characteristics such as radiodensity and chemical reactivity [4].

Diagnostic evaluation

Selecting the appropriate diagnostic tool to locate the foreign body depends on the time of presentation to the emergency department, the type of substance swallowed, and the severity of symptoms [4]. The imaging modality most commonly employed for initial screening is X-ray due to its convenience and accuracy [2,4]. Although CT has a sensitivity of 100% and specificity of 91%, it is usually reserved as a second-line modality for objects that are non-radiodense or too small and easily concealed behind soft tissue [11,12].

Treatment and intervention

About 80% of asymptomatic cases require no further intervention as the object passes on its own within four to five days, with the remaining cases requiring endoscopic intervention and/or surgery [4,13,14]. Conservative management involving close observation of the patient is preferred for objects that are <6 cm in size and <2.5 cm in diameter. Patients should consume a regular diet and are instructed to carefully observe their stool to ensure that the object has passed [4]. Esophagogastroduodenoscopy is indicated for objects lodged in the upper half of the gastrointestinal tract before the ligament of Treitz [4,15]. It is also used as an emergent intervention in the following three cases: ingestion of batteries, esophageal occlusion, and objects with sharp edges [3]. Surgical intervention is rare but reserved when the previously mentioned management strategies fail, that is, if the object is >6-7 cm in size, past the ligament of Treitz, and has not progressed in three days, or when complications such as perforations occur [15-18]. Factors that increase the risk of surgery include elevated differential blood count, previous surgeries, and a history of repeated ingestion [2,19].

## Conclusions

Repetitive ingestion of foreign objects in adults is most common among psychiatric populations and prisoners. In cases where a psychiatric history is apparent, prevention strategies such as limiting access to these items and psychotherapeutic intervention should be employed. A multidisciplinary team of specialists is required to both effectively manage patients’ symptoms and complications, if any arise, and to prevent the risk of recurrence. The method of intervention depends on the time and severity of presentation to the emergency department, with the three mainstays of treatment being conservative, endoscopic, and surgical.
